# Long-range connectomics

**DOI:** 10.1111/nyas.12271

**Published:** 2013-09-24

**Authors:** Saad Jbabdi, Timothy E Behrens

**Affiliations:** 1 FMRIB Centre, University of OxfordOxford, United Kingdom; 2 Wellcome Trust Centre for Neuroimaging Institute of Neurology, University College LondonLondon, United Kingdom

**Keywords:** brain connections, chemical tracers, tractography

## Abstract

Decoding neural algorithms is one of the major goals of neuroscience. It is generally accepted that brain computations rely on the orchestration of neural activity at local scales, as well as across the brain through long-range connections. Understanding the relationship between brain activity and connectivity is therefore a prerequisite to cracking the neural code. In the past few decades, tremendous technological advances have been achieved in connectivity measurement techniques. We now possess a battery of tools to measure brain activity and connections at all available scales. A great source of excitement are the new *in vivo* tools that allow us to measure structural and functional connections noninvasively. Here, we discuss how these new technologies may contribute to deciphering the neural code.

## Introduction

The importance of neural connections has been recognized since the beginnings of neuroscience,^1^ and theories of brain function have circuitry at their heart. In particular, the past few decades have seen a resurgence of interest in studying brain connections. This is in part due to the tremendous progress that has been achieved in measuring brain connections across all scales.

Methods for measuring detailed ultrastructural and microscopic organization of neuronal networks (individual axons, dendrites, and synapses) are now entering an industrial era.^2^–^8^ Tedious and error-prone manual delineation of intricate neural circuits over a few millimeters of tissue is currently being replaced with fast automated procedures that can process large sections of the brain. This type of high-throughput, high-fidelity data will constitute a vast wealth of connectivity information and will contribute to building a detailed understanding of neural circuits at microscopic scales.

At a larger scale, we also possess powerful tools for studying systems-level connections. In animal models, chemical tracers allow precise and accurate reconstruction of axonal bundles over their entire trajectories. In humans, modern imaging techniques allow *noninvasive* measurement of brain connections in living brains, and brought about the emerging field of *in vivo* connectomics.^9^ The ability to measure brain connections in living humans has generated much excitement and triggered large concerted efforts that attempt to push the limits of these methods. One notable example is the Human Connectome Project (HCP),^10^,^11^ a National Institutes of Health (NIH)-funded initiative that is aimed at charting the human macroconnectome in a large cohort of healthy adults using magnetic resonance imaging (MRI) and magnetoencephalography (MEG) technologies. A major focus of the HCP is to improve all aspects of data acquisition and processing to achieve much higher accuracy in building a macroconnectome than what can be achieved using current methods.^12^,^13^

How will these tools contribute to our understanding of brain function? Often, mechanisms of neural function are described in terms of local circuits, where the role of microconnectomics is unquestionable. For instance, microconnectomics provide statistical features and organizational principles of local connections^14^,^15^ that can guide computational models. Macroconnectomes, on the other hand, are only beginning to play such a mechanistic role. In this paper, we review the available tools for measuring large-scale connections, and we ask how knowledge of these long-range connections can contribute to cracking the neural code.

## Measuring long-range connections

**Figure 1:**
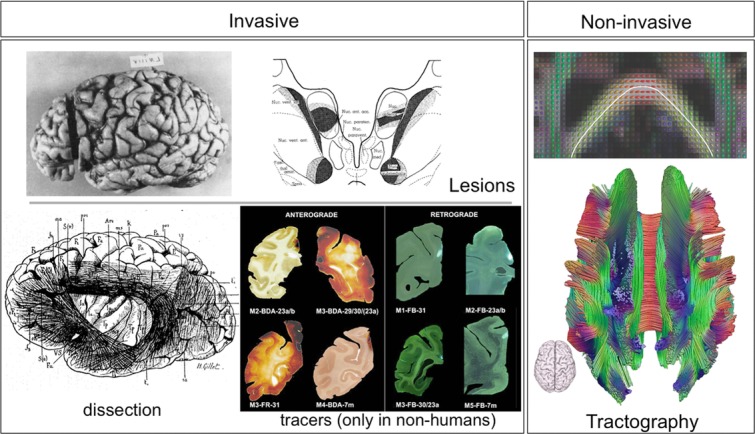
Available techniques for measuring anatomical connections in the brain. Lesion studies rely on Wallerian degeneration as a result of a brain lesion; the effects of the lesion can be seen postmortem at remote sites (here the thalamus) indicating the trajectories of white-matter projections (from Ref. 98). Postmortem dissections of white-matter connections date back to the 19th century (from Ref. 99). A multitude of tracers are available in animals. Shown here are example anterograde (biotinylated dextran amines, or BDA) and retrograde (Fast Blue fluorescent dye) tracers used to trace connections from the posterior cingulate cortex in macaques (from Ref. 100). The only available technique that is noninvasive is diffusion MRI tractography. The panel on the right shows how local estimates of fiber orientation, here using the diffusion tensor model, can serve to trace estimates of neural pathways. This allows reconstruction of major white-matter connections in the whole brain (top: figure from Ref. 31; bottom: image courtesy of Alexander Leemans).

Up until the end of the 20th century, all available tools for measuring long-range connections were invasive (Fig. 1). In addition, the most accurate tools, anterograde and retrograde tracers, were (and still are) only available in nonhuman animals. Recent advances in neuroimaging are providing a new set of tools that can be used in living humans.

### Tracers

Traditional methods for determining long-range connections between brain areas relied on lesion studies of axonal degradation. A revolution then occurred in the 1960s and 1970s, when a set of powerful and extremely versatile tract-tracing techniques was developed. These techniques rely on active, *in vivo* transport of compounds (e.g., proteins, amino acids, and viruses) along axons by means of cytoplasmic transport mechanisms, and are therefore extremely accurate. Tracers are injected into a source region, then after a certain amount of time, the brain is extracted, fixed, sectioned, and stained appropriately in order to detect traces of the compound at remote locations from the injection site.

A wide variety of tracers have been developed^17^ (Fig. 1). These tracers differ in properties that affect their transport speed and directionality (anterograde, retrograde, or both), and whether they can cross synapses. They also differ in how they react in histochemical or immunohistochemical reactions. Certain tracers can have fluorescence properties that alleviate the need for staining. This richness and variety of available compounds means that different tracer molecules can be used simultaneously on the same animal. Several connections can be traced at once, allowing the study of detailed circuits.^18^ An elegant demonstration of the power of such multiple tracer studies was shown by Lanciego *et al*.,^19^ who used a combination of retrograde and anterograde tracing to ask whether pallidal afferents that reach the substantia nigra innervate neurons that project to either the caudate or the putamen. Using differentially colored staining, overlapping areas between pallidonigral afferents and different subtypes of nigrostriatal projections could easily be identified.

Depending on the tracer that is used and on the staining process, it is possible to determine not only the precise termination point of axonal projections (e.g., cortical layer), but also sometimes reconstruct the entire trajectory of axonal pathways from source to target regions.^20^ In addition, modern developments in tract-tracing methods combine tracing long-range connections with detailed microanatomy.^21^ By using anterograde tracing of the projections combined with immunocytochemistry to identify the postsynaptic targets, it is possible to not only determine which regions are targeted (overall) by the tracers, but also to establish fine-grained connectivity such as whether synaptic contacts are made at the target region, and to determine neuronal subtypes that are targeted by long-range connections.^21^

Tracer studies continue to provide detailed pictures of systemic connectomes in many animal models. Of particular interest are studies of nonhuman primates anatomy.^22^–^26^ Compilations of many tracer studies in monkeys are beginning to provide quantitative data on large-scale connections throughout the cortex.^22^ These large-scale connectomes are a great source of information for studying organizational principles of brain connections and guiding electrophysiological recording and interpretation in monkey studies, and also constitute an estimate or at least an approximation, of the human large-scale connectome.

### Tractography

Tracers are only available in animals. As a result, and in contrast to the vast amount of connectivity data available in animal models, knowledge of human brain connectivity remains relatively poor.^27^

Studying brain connections in living humans has only been made possible following developments in diffusion magnetic resonance imaging (dMRI) in the mid to late 1990s.^28^ This noninvasive technique uses the dynamics of water molecular motion as a probe of tissue microstructure. Specifically, water motion in and around biological cells is hindered by cellular processes. The directionality of this hindrance is used as an indicator of tissue orientation. For example, in a region of tightly packed axons arranged along a common average orientation, water motion is less hindered along the axons than across them. By following the motion of water, it is possible to map the orientation(s) of fibers passing through each voxel of white matter. Long-range (>1 cm) connections can then be reconstructed using algorithmic approaches that integrate local estimates of fiber orientations over large distances: a technique called *tractography* or fiber tracking.^29^–^34^

The advent of *in vivo* dMRI tractography created a revolution in large-scale human connectomics. For the first time, we are able to virtually dissect large white-matter bundles in intact brains.^35^ Tractography has two striking advantages compared with chemical tract tracing. First, it is *in vivo* (although it can also be applied *ex vivo*^36^,^37^), and second, it allows us to measure connections in the whole brain at once. These two features of tractography opened a large number of new research possibilities. We can now measure brain structure and function on the same brains, and thus relate structural connections to brain function and behavior,^38^ analyze developmental pathways of structural connections,^39^ and relate structural connections to functional segregation,^40^ among many other possibilities that were unavailable two decades ago.

**Figure 2:**
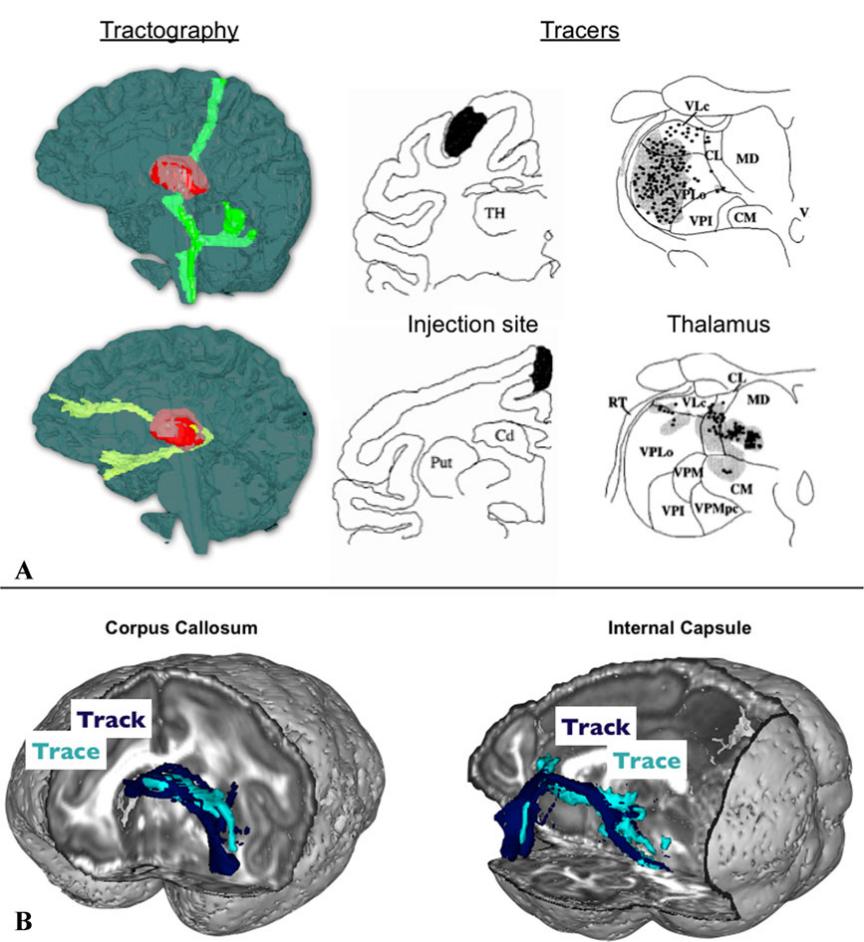
Two example comparisons between tractography and tracer results. (A) Connections traced from two locations in the thalamus using human dMRI tractography (left-hand side, modified from Ref. 74). Tracer studies in monkeys (right-hand side, modified from Ref. 101) shows that different thalamic regions contain traces of the injected dye depending on the cortical injection site. Comparing the two allows us to interpret the tractography result in terms of the location of the tractography seed relative to different thalamic nuclei. (B) Direct comparison of tractography and tracing of the same connections in the macaque brain. Shown here are two connections from the lateral orbitofrontal cortex traveling through the corpus callosum and the internal capsule, respectively, with a very good match between the two techniques. Modified from Ref. 47.

Noninvasiveness and whole-brainness also come at a cost: tractography is less accurate than chemical tracing. Although sensitive to microscopic features of the tissues, dMRI produces images at a much lower resolution than microscopy (>1 mm). Information about underlying cellular processes is averaged across tens of thousands of cells or axons. Therefore, only bulk connectivity can be assessed with this technique. Furthermore, dMRI measurements of tissue orientation are indirect; actual fiber organization is only inferred from water motion, a process that can be error prone, especially when the underlying axons within an imaging voxel lack organization.^41^,^42^ Improvements upon this promising measurement method are being carried out along several fronts. These include significant advances in imaging quality^13^ and algorithm developments,^12^–^46^ as well as validation and optimization using detailed comparisons of dMRI tractography and tracer studies in nonhuman primates.^47^ Figure 2 shows two examples of comparisons between the results of tracer studies and tractography in the monkey brain.

### In vivo *inference of structure from function*

Measurements of brain activity, as opposed to brain structure, can also be used as an alternative method for assessing connectivity. Resting-state functional MRI (rsfMRI; i.e., measurements of brain activity with MRI without a stimulus or an explicit task), has emerged as a powerful tool that provides information on network structure in the brain.^48^ Statistical dependencies in resting-state signal (or *functional connectivity*) between remote brain areas have been shown to reflect their anatomical connections.^49^,^50^ It is therefore thought that this type of measurement can be used, perhaps alongside dMRI tractography, to infer structural connections in the brain.^52^,^53^

Measurements of resting-state functional connectivity are also subject to their own biases and imperfections. Fortunately, the caveats of diffusion-based structural connectivity and resting-state functional connectivity are, to some extent, complementary. One example of this complementarity is evident when considering a caveat in tractography that is often referred to as the *distance bias*. Connectivity strength that is usually inferred from tractography tends to decrease with distance between the source and target areas. This reflects a decrease in the certainty of the orientation measurements, which is expected from the streamlining process.^31^–^55^ There is no such bias in rsfMRI, as the notion of functional connectivity does not rely on estimating the trajectories of the underlying axonal connections, although spatial autocorrelation in the rsfMRI signal can sometimes also induce a short-range bias in functional connectivity.

On the other hand, rsfMRI does not provide a complete picture of all anatomical connections in the brain. Clearly, anatomy constrains the statistical relations amongst neuronal time series, but this is a rather complex process and these statistical relationships are not a simple one-to-one mapping from anatomy. Statistical dependencies between connected areas may be transient (e.g., task related). Nonconnected regions may also exhibit dependencies owing to indirect projections, common input, or shared structured noise. While more sophisticated analysis methods^53^ may overcome some of these limitations, it is clear that the above-mentioned rsfMRI errors are not encountered in diffusion tractography. Therefore, the two techniques have complementary weaknesses. A multi-modal approach may ultimately allow us to capitalize on their strengths, and iron out their weaknesses.

## Relating long-range connections to brain function

While there is little debate that macroconnectomics is a key ingredient in understanding brain function at a systems level, it is useful to lay out specific examples of how macroconnectomes can be utilized in neuroscientific investigations. The remainder of this article highlights four broad research topics in neuroscience that will directly benefit from the availability of macroconnectomes.

### Bottom-up modeling

Large-scale neuronal network simulations are increasingly used as frameworks for studying links between anatomical connections and brain dynamics. An extreme example is the ambitious BlueBrain project,^56^ a colossal effort toward building a *virtual brain*, a large scale simulation on a supercomputer. Instead of summarizing small-scale activity with simplistic models of interacting excitatory and inhibitory cells, the BlueBrain project aims to model whole macrocolumns while accounting for the great variability in cell types and their chemical properties, with temporal dynamics simulated at high resolution (∼1 ms). Although such detailed bottom-up modeling promises to give us insights into local neuronal computations, we are still a long way from being able to run these types of simulations at the scale of whole brains.

**Figure 3:**
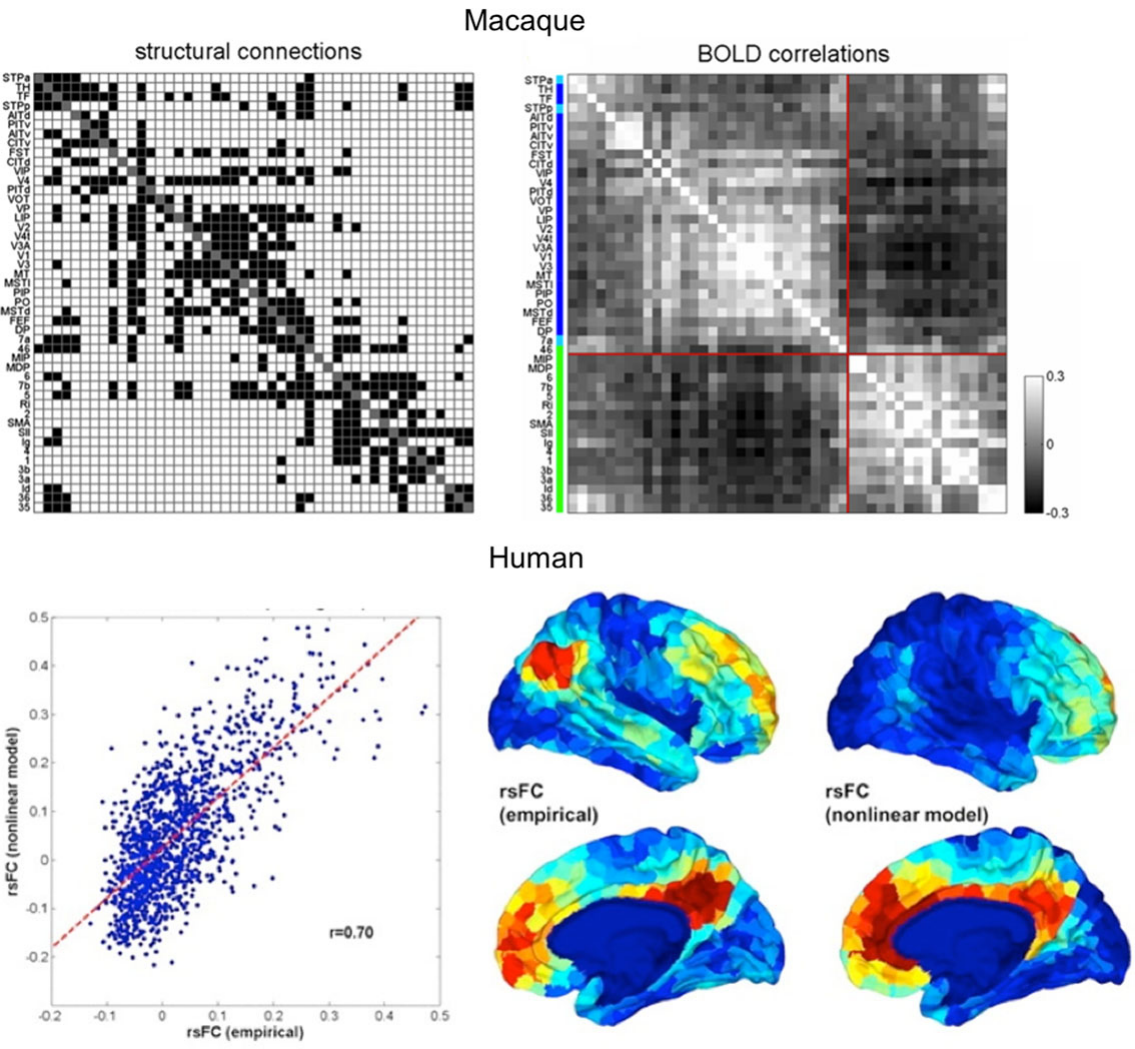
Bottom-up simulations of resting-state activity in macaques and humans. Top panel shows the anatomical network from CoCoMac, represented as a binary matrix of connections on the left, and the predicted fMRI functional connectivity on the right. The structure of the network induces the emergence of two anticorrelated subnetworks (modified from Ref. 64). Bottom panel shows the results of a similar modeling approach to human resting-state data. Structural connections were estimated using diffusion MRI tractography, and simulated fMRI functional connectivity was compared to empirical connectivity from a resting-state scan. The left-hand side shows the correlation between simulated and empirical whole-brain functional connectivity. The right-hand side shows a qualitative comparison between simulated and empirical functional connectivities of the posterior cingulate cortex. Modified from Ref. 58.

At a macroscopic scale, knowledge of brain circuitry can be utilized to build computational models of large-scale networks that can generate brain activity.^57^–^61^ Such computational models require the prespecification of a set of brain regions and precise knowledge of their connections. The online CoCoMac database^25^,^26^ has been used as a source of such information (Fig. 3). This compilation of tracer data in macaques was used in several studies where whole brain spatiotemporal dynamics were simulated using CoCoMac data as an estimate of the underlying anatomy.^59^–^64^ An example of the type of insight that these simulation studies can provide is given by Deco *et al*.^62^ This study used CoCoMac not only to model coupling strength between brain regions, but also to provide an estimate of conduction delay. The study showed how the structural features (delay and coupling) of a simplified macaque brain network can lead to the emergence of two sets of anti-correlated oscillators consistent with many experimental observations in humans and primates.

In humans, whole-brain tractography–derived connectivity has been used as a scaffold for simulating brain activity. For instance, Honey *et al*.^58^ used such an approach to test the extent to which we can predict resting-state fMRI measurements using systemic connectivity measures from dMRI tractography. Brain activity was generated using neural mass models of densely connected excitatory and inhibitory neurons. This local model was combined with large-scale coupling among brain areas where the coupling strength was directly proportional to structural measurements from dMRI tractography. The resulting ensemble activity was then turned into a hemodynamic signal that could be compared to empirical fMRI measurements (Fig. 3). Interestingly, this study found that brain activity over long time windows correlated strongly with the underlying anatomy.

A notable application of such network simulation approaches is to test disconnection hypotheses by simulating lesions in large-scale network models and observing alterations of brain activity as a result of these lesions.^65^,^66^

Dynamic causal modeling (DCM) is another example of a set of bottom-up computational models that combine large-scale connectivity with local-scale dynamic models.^67^ DCMs typically consider circuits that consist of a small set of brain regions (3–10) and seek to model the influence that each region exerts on the other regions of the network via large scale reciprocal connections. DCM for electrophysiological data, such as electroencephalography, emphasizes detailed circuit modeling, thus enabling inferences on both large-scale interactions and local-scale properties of microcircuits.^68^–^69^ DCMs typically require setting up an underlying anatomical model that constrains the possible routes of activity propagation between brain regions. Such a model may come from a priori anatomical knowledge, although use of dMRI tractography to constrain the anatomical model has also been suggested.^52^–^54^

### Functional specialization

Ever since the times of Broca and his famous patient Tan, there has been overwhelming evidence for functional specialization in the brain. An important question in systems neuroscience is how to derive a subdivision of the brain that reflects this functional specialization. Neuroanatomists of the 20th century tackled this problem using postmortem histological tools that measure cytoarchitecture, myeloarchitecture, and more recently, receptoarchitecture.^70^ Subsequent studies of brain function have shown that histological features are overall good predictors for functional segregation.^71^ On the other hand, macroscopic landmarks, such as cortical folds, are not always good indicators for transitions between functional regions.^72^ Therefore, postmortem cytoarchitectonic subdivisions cannot easily be transferred into studying living brains.

**Figure 4:**
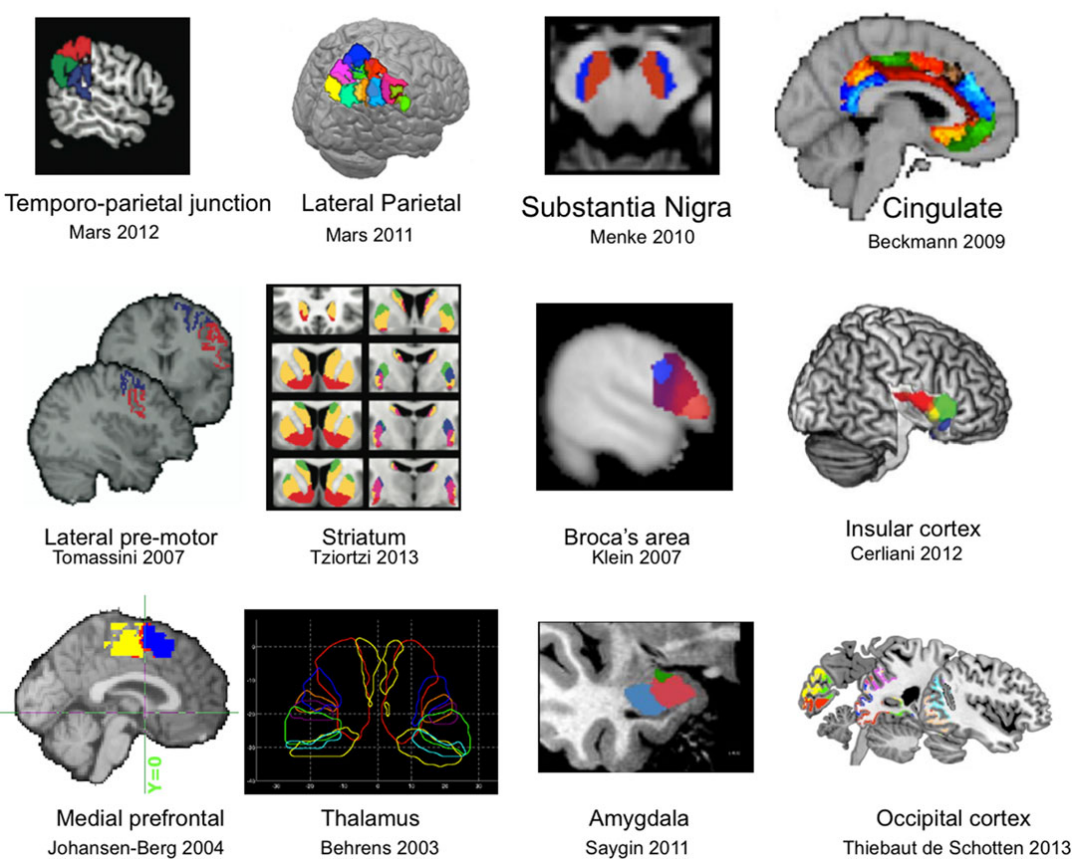
Examples of tractography-based parcellations of cortical and subcortical regions in humans. From left to right and top to bottom Refs. 85, 84, 102, 81, 87, 77, 103, 104, 83, 74, 105, 106, with permission.

An alternative approach is to use connectivity. The extrinsic connections of a cortical area impose constraints on the type of information that an area can send or receive, and thus to some extent determine its putative function.^73^ Exploiting this principle, both tractography and rsfMRI have been used to segregate gray matter according to the route of white-matter projections (extrinsic connectivity) or coherence in brain activity, respectively, both in the subcortex^74^–^79^ and the neocortex^77^–^87^ (Fig. 4). Many of these studies have shown a remarkable degree of similarity between regional borders identified using tractography and various other methods, including histological atlases, functional MRI activations, or other structural imaging modalities.

In addition to finding borders between separable regions in the brain, macroscopic connections can also help us to understand the computations and internal organization of brain regions. For instance, sensory cortices are laid out topographically, and computations within these regions are therefore spatially organized on the 2D cortical surface. Long-range connections likely reflect this topographic organization to some extent^88^ and may therefore be used to further characterize the internal organization of functional regions.

### Functional integration

The flip side of functional specialization is functional integration, which emphasizes how brain regions interact and influence one another. Graph theory has a central role in studying functional integration. A graph, or network, is an abstract but relatively familiar object that consists of a set of nodes and edges between these nodes. This makes it a natural mathematical description of the brain in relation to regions (nodes) and the physical connections between them (edges). Once such abstraction has been adopted, a large number of graph-theoretical concepts and measures become available for studying and quantifying the topology of the graph.^89^ Many of these measures have been applied in other types of biological or social networks.

Rather than focus on the details of specific brain connections, network measures attempt to distil principles of organization and provide a set of statistics that reflect certain network characteristics. Some of these characteristic features reflect the degree to which brain regions are segregated, integrated, or clustered, highlighting, for instance, putative hubs. Other measures quantify efficiency of information propagation, establishing links between network structure and dynamics.

Numerous studies used network theory to quantify macroconnectomes derived from various types of MRI data. While there is still debate as to how various stages of data processing affect network measures, a converging picture is starting to emerge. For instance, certain brain regions of the parietal and frontal cortices have consistently been identified as central hubs connected to a structural core.^90^

### Probing circuits

While a global picture of the brain macroscopic network is useful to derive general principles of organization and understand global dynamics of brain activity, scientists are often interested in studying specific brain subsystems related to specific behaviors. Such studies are of course not feasible without the ability to measure both brain function and connections in the same animal or individual.

The combination of diffusion MRI and tractography allow not only the reconstruction of major white-matter connections, but also provide measurements of microscopic and macroscopic features of those connections. For instance, certain aspects of diffusion, such as its anisotropy, are thought to indicate axonal integrity at a microscopic level. Tract volume is another (macroscopic) measure that is often used to estimate the prominence of certain connections. Together, these micro- and macroscopic features have been used in numerous studies of brain connections in diseases,^91^ development,^39^ aging,^92^ and a number of different behaviors such as visuospatial attention,^93^ language,^94^ cognitive control,^95^ and skill learning.^38^ Often, these studies proceed in an exploratory fashion, asking which among all measureable brain connections relate to behavior or a disease process. The availability of whole-brain connectivity afforded by tractography is therefore key to such studies.

In contrast, other studies can be guided by specific hypotheses. For instance, Aron *et al*. triangulated a cognitive-control network composed of the inferior prefrontal cortex, the subthalamic nucleus and the presupplementary motor area.^96^ Their idea was to use brain activity measurements from fMRI to determine a network of regions involved in response inhibition. They then showed that each node in the network formed connections with the other two, supporting the idea of a three-way functional–anatomical network.

Another elegant demonstration of hypothesis-driven investigation of anatomy versus function is a study by Saalmann *et al*.,^97^ who were investigating the role of the pulvinar nucleus of the thalamus in selective attention. Using simultaneous recording of electrical activity in interconnected areas of the thalamus and cortex in macaque monkeys, they were able to show that the pulvinar synchronizes activity between cortical areas according to attentional allocation. In a nice demonstration of combining structural and functional measurement methods, the study used dMRI tractography to locate connected subregions of the pulvinar and cortex as a guide for electrode placement.

It is interesting to note the use of tractography in the above-mentioned study, despite the fact that the study was done in monkeys, where tracers are available and presumably more accurate than tractography. However, by using tractography, Saalman *et al*. indirectly highlight the striking advantages that tractography has over the much more accurate tracer methods available in macaques. Using tracers would have required gathering data across several animals and extrapolating the results to the animals studied with electrophysiology. Tractography provides the required connections in the same animals.

## Conclusion

Our toolbox for measuring brain connections is filling up with tools that are constantly increasing in quality and accuracy. Large-scale connections can now be measured, although with some degree of uncertainty, in living brains, and we can therefore relate connections to brain dynamics and to behavior. At the same time, tremendous progress is being made in postmortem measurement of connections at microscopic scales, with a view to one day being able to map all such connections in the entire brain. The future will perhaps see these two worlds of long-range and local connections converge into a multiscale view of brain connectomics.

## Conflicts of interest

The authors declare no conflict of interest.
